# NANOG regulates epithelial–mesenchymal transition via AMPK/mTOR signalling pathway in ovarian cancer SKOV‐3 and A2780 cells

**DOI:** 10.1111/jcmm.17557

**Published:** 2022-09-16

**Authors:** Hee Yun, Gwan Hee Han, Julie Kim, Joon‐Yong Chung, Jae‐Hoon Kim, Hanbyoul Cho

**Affiliations:** ^1^ Department of Obstetrics and Gynecology, Gangnam Severance Hospital Yonsei University College of Medicine Seoul Korea; ^2^ Department of Obstetrics and Gynecology Kyung Hee University Hospital at Gangdong Seoul Korea; ^3^ Weill Cornell Medical College New York New York USA; ^4^ Molecular Imaging Branch, Center for Cancer Research, National Cancer Institute National Institutes of Health Bethesda Maryland USA; ^5^ Department of Obstetrics and Gynecology Yonsei University College of Medicine Seoul Korea; ^6^ Institute of Women's Life Medical Science Yonsei University College of Medicine Seoul Korea

**Keywords:** epithelial–mesenchymal transition, mTOR, NANOG, ovarian cancer, pAMPK

## Abstract

NANOG engages with tumour initiation and metastasis by regulating the epithelial–mesenchymal transition (EMT) in epithelial ovarian cancer (EOC). However, its role in association with pAMPKα, and its clinical significance in EOC have not been elucidated even though AMPK is known to degrade NANOG in various human cancers. Hence, we investigated the role of pAMPKα and its association with NANOG as potential prognostic biomarkers in EOC. Both NANOG and pAMPKα expression were significantly overexpressed in EOCs comparing nonadjacent normal epithelial tissues, benign tissues, and borderline tumours. NANOG overexpression was significantly associated with poor disease‐free survival (DFS) and overall survival (OS), whereas pAMPKα overexpression was associated with good DFS and OS. Importantly, multivariate analysis revealed that the combination of high NANOG and low pAMPKα expression was a poor independent prognostic factor for DFS and was associated with platinum resistance. In ovarian cancer cell lines, siRNA‐mediated NANOG knockdown diminished migration and invasion properties by regulating the EMT process via the AMPK/mTOR signalling pathway. Furthermore, treatment with AMPK activator suppressed expression of stemness factors such as NANOG, Oct4 and Sox2. Collectively, these findings established that the combination of high NANOG and low pAMPKα expression was associated with EOC progression and platinum resistance, suggesting a potential prognostic biomarker for clinical management in EOC patients.

## 1 INTRODUCTION

Based on data from global cancer statistics (Global Cancer Incidence, Mortality and Prevalence [GLOBOCAN]), ovarian cancer is the 8th most common cancer among women globally, with an estimated 313,000 new cases and 207,000 deaths in 2020.^1^ Despite improvements in treatment, such as target therapy or immunotherapy,^2^ the prognosis of patients with epithelial ovarian cancer (EOC) remains poor due to a lack of effective clinical screening methods, resulting in diagnoses at advanced stages.^3^ As such, most EOC patients have metastatic disease at the time of diagnosis.^4^ Thus, it is crucial to investigate biomarkers for the early detection of EOC as well as their metastasis to reduce disease mortality and platinum resistance.

Epithelial–mesenchymal transition (EMT) is essential for the initiation of metastasis for cancer progression. Recent studies have suggested that ovarian cancer cells acquire mesenchymal traits and lose epithelial traits when they attain the ability to invade.^5^ EMT‐positive status has been correlated with poor progression free survival and overall survival.^6^ Furthermore, in metastatic serous ovarian carcinoma effusions, several proteins related to EMT, including vimentin and ZEB1, have been found to be markers of a poor chemotherapy response.^7^

Recent studies have highlighted a link between EMT and the properties of cancer stem cells (CSCs).^8^ As a central stemness‐associated transcription factor, NANOG regulates the fundamental properties of CSCs, such as cell proliferation, cell cycle, self‐renewal, EMT, tumorigenicity, and chemoresistance.^9^, ^10^, ^11^ In ovarian cancer, the androgen receptor contributes to the function of NANOG, which subsequently promotes ovarian CSC maintenance. Samples of metastatic foci as well as ovarian cancer cell lines with features associated with metastasis have both been found to have high NANOG expression.^12^ Additionally, in patients with colorectal cancer, breast cancer, and ovarian serous carcinoma, NANOG expression has been linked to poor prognosis.^13^, ^14^, ^15^ As EMT and the metastatic cascade are highly energy‐consuming, the balance between ATP consumption and production could be critical for the motile and invasive capacities of cancer cells. AMP‐activated protein kinase (AMPK), known as a master regulator of energy homeostasis, is pivotal in cancer metastasis as well as cancer cell metabolism.^16^, ^17^ Numerous studies have shown that AMPK suppresses EMT in various cell types, such as tubular epithelial cells,^18^, ^19^ breast cancer cells,^20^ lung adenocarcinoma cells^21^ and bronchial epithelial cells.^22^ Moreover, increased expression of phosphorylated AMPKα (pAMPKα) in solid tumours, including breast, lung, and gastric cancers, has been shown to be associated with both prognosis and tumour grade.^23^, ^24^, ^25^ Given that AMPK is associated with cancer metastasis, we hypothesized that NANOG modulates AMPK signalling to regulate the EMT process and promote ovarian cancer metastasis.

In this study, we investigated the clinicopathological features and prognostic significance of NANOG and pAMPKα in ovarian cancer. Additionally, we identified a novel mechanism by which NANOG increases the metastatic potential of ovarian cancer cells by regulating the AMPK/mTOR pathway, which might foster new therapeutic strategies for the treatment of ovarian cancer.

## 2 MATERIALS AND METHODS

### 2.1 Patients and tumour specimens

A total of 417 tissue samples (212 EOCs, 52 borderline tumours, 83 benign tumours, and 70 nonadjacent normal ovarian epithelia) obtained from patients who underwent primary cytoreductive surgery at Gangnam Severance Hospital between 1996 and 2012 and the Korean Gynecologic Cancer Bank were included in the study. The clinical information of patients, including age, International Federation of Gynecology and Obstetrics (FIGO) stage, histology based on the World Health Organization grading system, surgical procedure, response to platinum‐based chemotherapy, level of cancer antigen 125 (CA‐125), survival time and survival status, were collected by reviewing medical records and pathology reports. All patients were treated with maximal debulking surgery without residual disease, followed by adjuvant chemotherapy with paclitaxel/carboplatin. DFS was evaluated from the date of surgery to the period of recurrence/progression or the time of the last follow‐up visit assessed by Response Evaluation Criteria in Solid Tumors (RECIST; version 1.1) based on response to therapy by spiral computed tomography (CT) or positron emission tomography (PET) – CT.^26^ OS was assessed from the period from the date of surgery to either the patient's death or the date of last contact for living patients. The platinum resistance ovarian cancer was defined as disease recurrence within 6 months of complete of the first line platinum‐based chemotherapy. All tumour tissues were histologically examined by one gynaecologic pathologist, and all biological samples were collected after obtaining informed consent from the participants, according to the guidelines of the Institutional Review Board (IRB) of Gangnam Severance Hospital (IRB No. 3‐2020‐0377).

### 2.2 Tissue microarray and immunohistochemistry

TMA, which was constructed in previous studies, was used in this study.^27^, ^28^ The TMA blocks were cut to 5‐μm thickness with a rotary microtome. After sectioning, the TMA sections were deparaffinized with xylene and dehydrated in serially graded ethanol to distilled water. Then, antigen retrieval was performed by incubating TMA sections using a steam pressure cooker (Pascal; Dako, Carpinteria, CA) in heat‐activated antigen retrieval buffer at pH 6.0 (Dako) for anti‐NANOG and at pH 7.8 for anti‐phospho AMPKα (Thr172). The sections were treated with 3% H_2_O_2_ solution in methanol for 10 min to block the endogenous peroxidase activity. After rinsing the slides, they were stained with an anti‐NANOG antibody (rabbit antibody, clone#4903S, 1:200; Cell signalling Technology, Inc., Danvers, MA) for 1 hour and anti‐pAMPKα antibody (rabbit antibody, clone#2535, 1:52; Cell signalling Technology, Inc.) for 32 min at room temperature. Subsequently, the antigen–antibody reactions were visualized by using Envision^+^ Dual Link System‐HRP (Dako) and DAB^+^ (3, 3′‐diaminobenzidine; Dako) for 10 min. The stained sections were dehydrated and counterstained with haematoxylin and mounted in Faramount aqueous mounting medium (Dako). Appropriate negative and positive controls were included.

### 2.3 Evaluation of IHC staining

The stained TMA sections were scanned using a high‐resolution optical scanner (NanoZoomer 2.0 HT; Hamamatsu Photonics K.K., Hamamatsu City, Japan) at a 20× objective magnification (0.5 μm resolution). The scanned sections were analysed with Visiopharm software, version 4.5.1.324 (Hørsholm, Denmark). Brown staining intensity was scored on a scale by intensity from 0 to 3 (0 = negative, 1 = weak, 2 = moderate, and 3 = strong) and percentage of the cytoplasm‐stained tumour cells (range, 0–100) was obtained by using a predefined optimized algorithm. The overall histoscore was calculated by multiplying the percentage of the positive cells and intensity score (score range: 0–300).^29^

### 2.4 Cell culture and reagents

The human ovarian cancer cell lines, SKOV‐3 and A2780 were purchased from the American Type Culture Collection (ATCC, Manassas, VA, USA) and the European Collection of Cell Cultures (ECACC, Salisbury, United Kingdom), respectively. The cells were cultured in RPMI 1640 supplemented with 10% foetal bovine serum and 1% penicillin/streptomycin in an atmosphere of 5% CO_2_ at 37°C. AICAR, allosteric activator of AMPK, was purchased from Cell Signalling Technology. Compound C, selective and ATP‐competitive AMPK inhibitor, was purchased from Selleck Chemicals (Houston, TX, USA).

### 2.5 siRNA transfection

Specific small interfering RNAs (siRNAs) for NANOG and control siRNA (siControl) were purchased from Bioneer (Daejeon, Korea). The siRNA sequences were as follows: NANOG_#1, 5′‐AGUGUUUCAAUGAGU‐3′ (sense), 5′‐ACUCAUUGAAACACU‐3′ (antisense); NANOG_#2, 5′‐UCUCGUAUUUGCUGC‐3′ (sense), and 5′‐GCAGCAAAUACGAGA‐3′ (antisense). siRNA pools for NANOG were purchased from Santa Cruz Biotechnology (Santa Cruz, CA, USA). siRNA was transfected into 6‐well plates at a dose of 100 pmol per well using Lipofectamine® RNAiMAX Reagent (Invitrogen, Gaithersburg, MD, USA) according to the manufacturer's instructions.

### 2.6 Western blot analysis

Cells were harvested and lysed with cell lysis buffer (Cell Signalling Technology, Danvers, MA, USA) containing PMSF and NaF. Proteins from cell lysates were resolved by SDS‐PAGE and transferred to a nitrocellulose membrane. Antibodies against α‐actinin (sc‐17829) and p70S6K (sc‐8419) were purchased from Santa Cruz Biotechnology (Santa Cruz, CA, USA). Antibodies against NANOG (#3580), ZEB1 (#3396), vimentin (#5741), N‐cadherin (#13116), phospho‐AMPK^Thr172^ (#2535), AMPK α (#2532), phospho‐mTOR^Ser2448^ (#5536), mTOR (#2983), and phospho‐p70S6K^Thr389^ (#9234) were purchased from Cell Signalling Technology. Immunoreactive bands were visualized using enhanced chemiluminescence reagents (Thermo Fisher Scientific, Waltham, MA, USA).

### 2.7 Boyden chamber assay

To examine cell invasion, 48‐well micro chemotaxis chambers (Neuro Probe, Gaithersburg, MD, USA) were used. Culture medium containing 10% FBS was added to the bottom chambers, which were then covered with Matrigel (BD Biosciences, San Jose, CA) coated membranes (#PFB8; Neuro Probe). At 48 h post‐transfection, siRNA‐transfected cells (1 × 10^5^ cells/50 μl of medium containing 0.05% FBS) were seeded in upper chambers. After 48 h, the membranes were fixed and stained using Diff‐quik solution (Sysmex, Kobe, Japan). The uninvaded cells were removed from the upper surface of the membrane, and the invading cells were counted in six random high‐power fields per filter using an Axio Imager M2 microscope (Carl Zeiss, Thornwood, NY, USA). Each experiment was repeated three times.

### 2.8 Real‐time quantitative RT‐PCR

Total RNA was isolated using the AccuPrep® Universal RNA Extraction Kit (Bioneer), and cDNA was synthesized using AccuPower® RocketScript™ RT PreMix (Bioneer) according to the manufacturer's protocol. Real‐time quantitative PCR was performed using TOPrealTM qPCR 2X PreMIX (SYBR Green with high ROX; Enzynomics, Daejeon, Korea) on an Applied Biosystems 7300 real‐time PCR system (Applied Biosystems, Foster City, CA). The reaction conditions were as follows: preincubation at 94°C for 10 min, followed by 40 cycles of 94°C for 10 s, 60°C for 15 s, 72°C for 15 s, and a melting curve program, with the temperature rising from 60 to 95°C. The comparative cycle threshold (2^−∆∆Ct^) method was used to calculate the relative mRNA expression levels, and the β‐actin gene was used as the endogenous control for normalization. Primers were purchased from Bioneer; NANOG 5′‐CCATCCTTGCAAATGTCTTCTG‐3′ (forward); 5′‐CTTTGGGACTGGTGGAAGAATC‐3′ (reverse), OCT4 5′‐GTGGAGGAAGCTGACAACAAT‐3′ (forward); 5′‐AATTCTCCAGGTTGCCTCTCACT‐3′ (reverse), SOX2 5′‐CGAGATAAACATGGCAATCAAAAT‐3′ (forward); 5′‐AATTCAGCAAGAAGCCTCTCCTT‐3′ (reverse), β‐actin 5′‐CATCCGCAAAGACCTGTACGCCAAC‐3′ (forward); and 5′‐ATGGAGCCGCCGATCCACA‐3′ (reverse). Each experiment was repeated three times.

### 2.9 Statistical analysis

The Mann–Whitney test or Kruskal–Wallis test was performed for statistical analysis of pAMPKα and NANOG expression levels, as appropriate. The Kaplan–Meier method was used to analyse the overall survival (OS) and disease‐free survival (DFS) curves by subgrouping NANOG as high or low expression group and pAMPK as low or high expression group by using the optimal cut‐off point calculated by “MaxStat” package of R software.^30^ In addition, the Cox proportional hazard model was used to calculate hazard ratios and confidence intervals (CIs) in both univariate and multivariate models. Statistical analyses were performed by using SPSS version 25.0 (SPSS Inc., Chicago, IL). Statistical significance was set at *p* < 0.05.

## 3 RESULTS

### 3.1 pAMPK and NANOG protein expression in ovarian cancer

**FIGURE 1:**
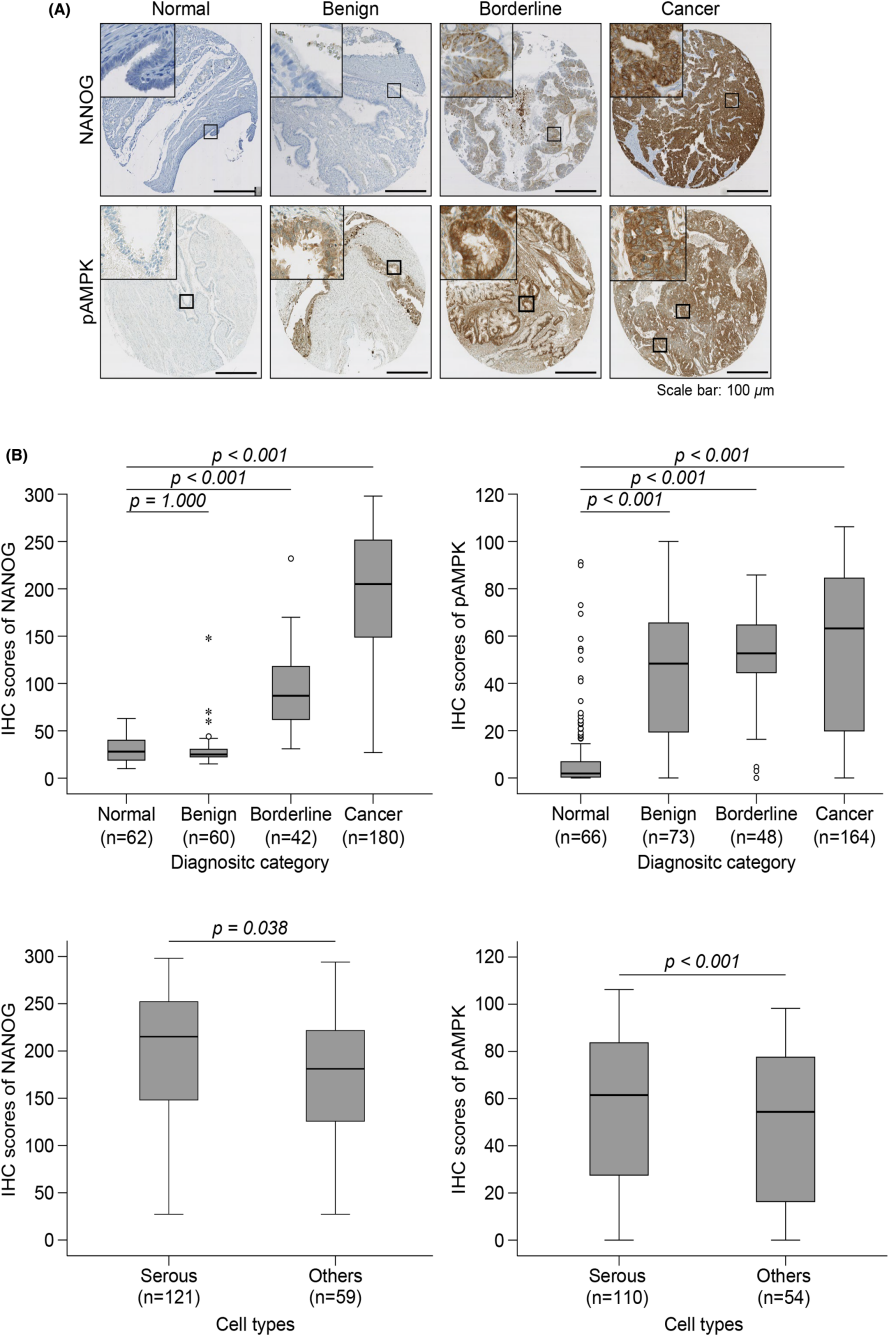
High NANOG or pAMPKα expression was observed in EOC and its clinicopathological significance in EOC patients. (A) Representative immunohistochemical stating images of NANOG or pAMPKα in adjacent normal epithelial tissues, benign, borderline tumours and EOCs. (Scale bar 100 μm) (B) Boxplots of NANOG or pAMPKα according to clinicopathological characteristics.

To investigate the clinical implications of NANOG and pAMPK in EOC, we performed IHC with TMAs from 212 EOCs, 52 borderline tumours, 83 benign tumours, and 70 nonadjacent normal epithelial tissues. However, due to loss of spots while sectioning and spots containing less than 100 tumour cells were excluded. Finally, 180 EOCs (121 serous, 26 mucinous, 8 clear, 25 endometrioid), 42 borderline tumours (35 serous, 7 mucinous), 60 benign tumours (43 serous, 10 mucinous, 7 endometriosis), and 62 nonadjacent normal epithelial tissues for NANOG and 164 EOCs (110 serous, 25 mucinous, 6 clear, 23 endometrioid), 48 borderline tumours (38 serous, 10 mucinous), 73 benign tumours (54 serous, 15 mucinous, 4 endometriosis), and 66 nonadjacent normal epithelial tissues for pAMPK were interpretable for evaluation of the association between NANOG or pAMPK and clinicopathological characteristics in EOC patients. As previously reported,^31^ we also observed abundant NANOG expression in cytoplasm, and significantly higher expression in EOCs comparing borderline, benign and nonadjacent epithelial tissues (*p* < 0.001; Table 1, Figure 1A,B). In case of pAMPK, we observed abundant expression in cytoplasm, and significantly higher expression in EOCs comparing nonadjacent epithelial tissues (*p* < 0.001; Table 1, Figure 1A,B). In addition, both NANOG and pAMPK were significantly associated with serous cell type (*p* = 0.038, *p* < 0.001; Table 1, Figure 1B, respectively). However, when looking at FIGO stage, NANOG was associated with advanced FIGO stage whereas pAMPK was significantly associated with early FIGO stage. Additionally, high NANOG expression was associated with advanced tumour grade (*p* < 0.001; Table 1).

**TABLE 1:** Expression of NANOG and pAMPK in relation to clinicopathological characteristics in IHC analysis

	No.	NANOG		No.	pAMPK	
		Mean score (95% CI)	*p* value		Mean score (95% CI)	*p* value
All study subjects	363	125.3 [115.6–135.1]		367	41.4 [38.1–44.8]	
Diagnostic category			<0.001			< 0.001
Normal	62	29.45 [26.06–32.84]		66	8.89 [5.86–11.91]	
Benign	60	29.83 [25.34–34.33]		73	45.30 [38.7–51.9]	
Borderline	42	93.02 [79.32–106.73]		48	49.87 [43.78–55.96]	
Cancer	180	190.79 [180.36–201.23]		164	55.17 [50.17–60.16]	
FIGO stage			0.116			0.007
I–II	53	171.64 [151.98–191.3]		52	65.1 [55.52–74.68]	
III–IV	127	190.98 [177.6–204.37]		112	53.8 [47.58–60.01]	
Cell type			0.038			< 0.001
Serous	121	193.38 [180.09–206.67]		110	63.57 [58.5–68.63]	
Others	59	168.69 [148.97–188.42]		54	49.82 [44.94–54.69]	
Tumour grade			<0.001			0.324
Well/Moderate	78	169.22 [151.96–186.47]		75	55.20 [47.74–62.66]	
Poor	86	206.72 [192.83–220.61]		78	60.07 [53.24–66.89]	
CA125			0.221			0.131
Negative	25	167.40 [136.14–198.66]		23	65.35 [50.80–79.91]	
Positive	153	187.28 [175.31–199.25]		139	53.48 [47.63–59.33]	
Chemosensitivity			0.418			0.212
Sensitive	153	184.69 [172.64–169.73]		136	57.11 [51.25–62.96]	
Resistant	13	202.31 [158.64–245.98]		12	44.20 [25.63–62.79]	

Abbreviations: CI, confidence interval; FIGO, International Federation of Gynaecology and Obstetrics.

### 3.2 Prognostic significance of pAMPK and NANOG expression

**FIGURE 2:**
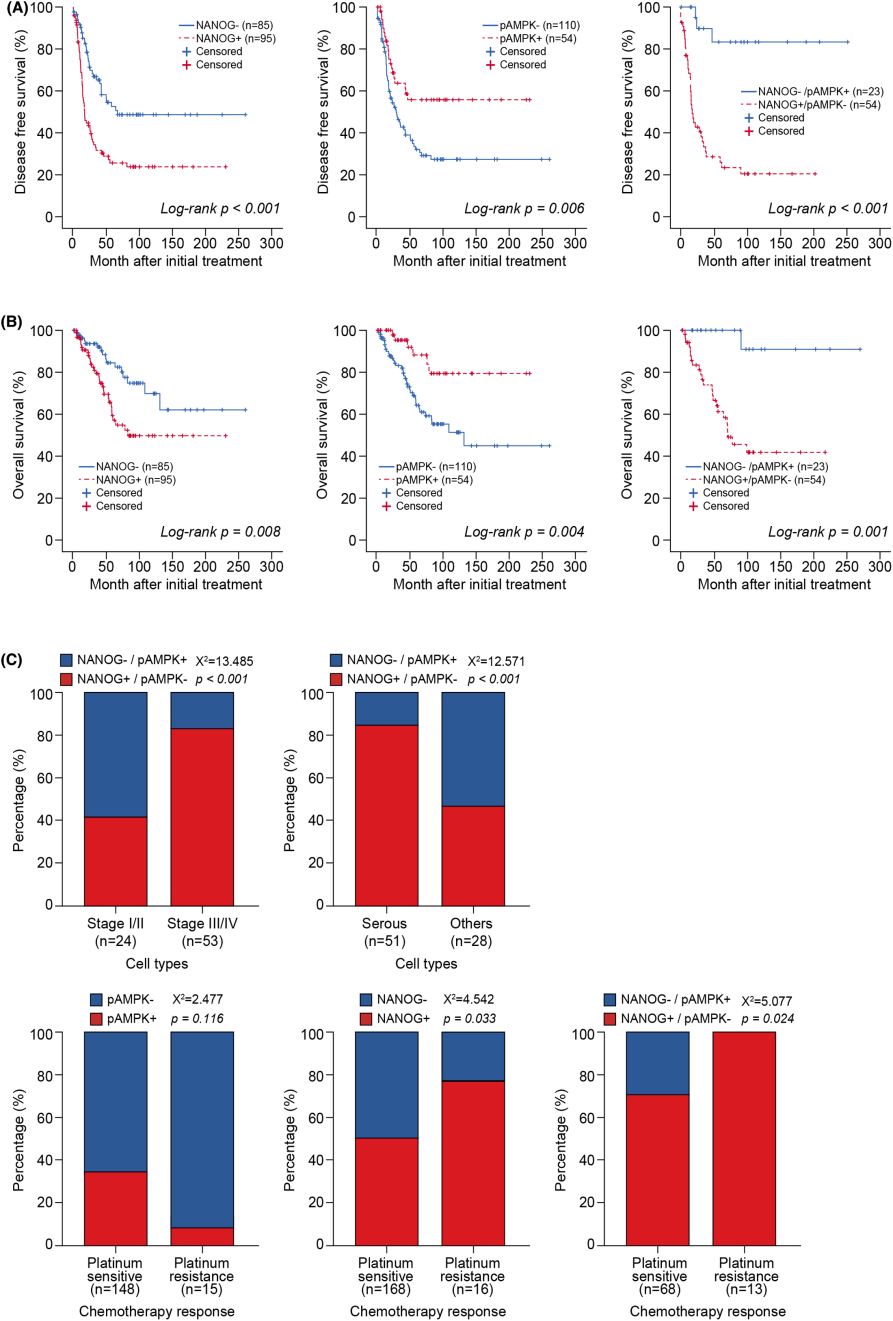
Kaplan–Meier survival curve of NANOG or pAMPKα expression in EOC. (A) Overall survival of EOC patients was analysed according to NANOG, pAMPKα, or combination of NANOG and pAMPKα expression. (B) Disease‐free survival of EOC patients with NANOG, pAMPKα, or combination of NANOG and pAMPKα expression pattern. (C) Clinicopathological characteristics of EOC patients with various NANOG and pAMPKα expression.

Next, we evaluated the prognostic significance of NANOG and pAMPK expression in EOC patients via a Kaplan–Meier curve, which demonstrated that the patients with higher NANOG expression (NANOG^high^) were significantly associated with poor DFS and OS compared to lower expression of NANOG (NANOG^low^) in EOC patients (*p* < 0.001, *p* = 0.008; Figure 2A,B). Meanwhile, higher pAMPK (pAMPK^high^) was significantly associated with better DFS and OS compared with lower pAMPK (pAMPK^low^) expression in EOC patients (*p* = 0.006, *p* = 0.004; Figure 2A,B). Next, we aimed to identify the prognostic value of combining NANOG and pAMPK (NANOG^high^/pAMPK^low^ vs NANOG^low^/pAMPK^high^) instead of a single biomarker, since previous studies have reported that the activation of pAMPK deregulates the stability of NANOG and causes its degradation.^32^ The survival analysis revealed that NANOG^high^/pAMPK^low^, compared with NANOG^low^/pAMPK^high^, was significantly correlated with poor DFS and OS in EOC patients (*p* = 0.001 and *p* < 0.001, respectively; Figure 2A,B). Furthermore, Cox proportional univariate analyses and multivariate analyses adjusted for FIGO stage, cell type, tumour grade and CA 125 showed that the combined biomarker, NANOG^high^/pAMPK^low^, was a stronger predictive biomarker for DFS in EOC than a single biomarker (HR = 5.29, [95% CI: 1.45–19.34], *p* = 0.012; Table 2).

**TABLE 2:** Univariate and multivariate analyses of the associations between prognostic variables and disease‐free survival in epithelial ovarian cancer

	Disease‐free survival hazard ratio [95% CI], *p* value	
	Univariate	Multivariate
FIGO stage (III–IV)	6.42 [3.33–12.39], <0.001	4.36 [2.05–9.26], < 0.001
Cell type (serous)	0.33 [0.2–0.55], <0.001	0.47 [0.25–0.88], 0.018
Tumour grade (poor)	1.95 [1.28–2.97], 0.002	1.36 [0.86–2.14], 0.187
CA125^+^ (>35 U/ml)	2.39 [1.2–4.74], 0.013	0.89 [0.39–2.03], 0.79
Age (>50)	1.58 [1.06–2.35], 0.024	1.18 [0.76–1.82], 0.467
NANOG^high^ ^a^	2.33 [1.53–3.53], <0.001	1.93 [1.22–3.06], 0.005
pAMPK^low^ ^b^	2.00 [1.21–3.31], 0.007	1.19 [0.7–2.05], 0.518
NANOG^high^/pAMPK^low^	9.24 [2.84–30.1], 0.001	5.29 [1.45–19.34], 0.012

Abbreviations: CI, confidence interval; FIGO, International Federation of Gynecology and Obstetrics; LN, lymph node; NA, not applicable.

^a^ Cut‐off value of NANOG^high^ is over 189 of IHC score.

^b^ Cut‐off of pAMPK^low^ is less than 79.92 of IHC score.

As NANOG^high^/pAMPK^low^ was revealed to be a more valuable prognostic biomarker for DFS in EOC patients, we further validated the clinicopathological characteristics, with the results showing that that it was associated with advanced FIGO stage and serous cell type (both *p* < 0.001; Figure 2C). Most importantly, the combination of NANOG and pAMPK (NANOG^high^/pAMPK^low^) showed a higher prognostic value for chemotherapy‐resistance than single protein expression (*p* = 0.039; Figure 2C).

### 3.3 Suppression of NANOG expression inhibited EMT in ovarian cancer cells

**FIGURE 3:**
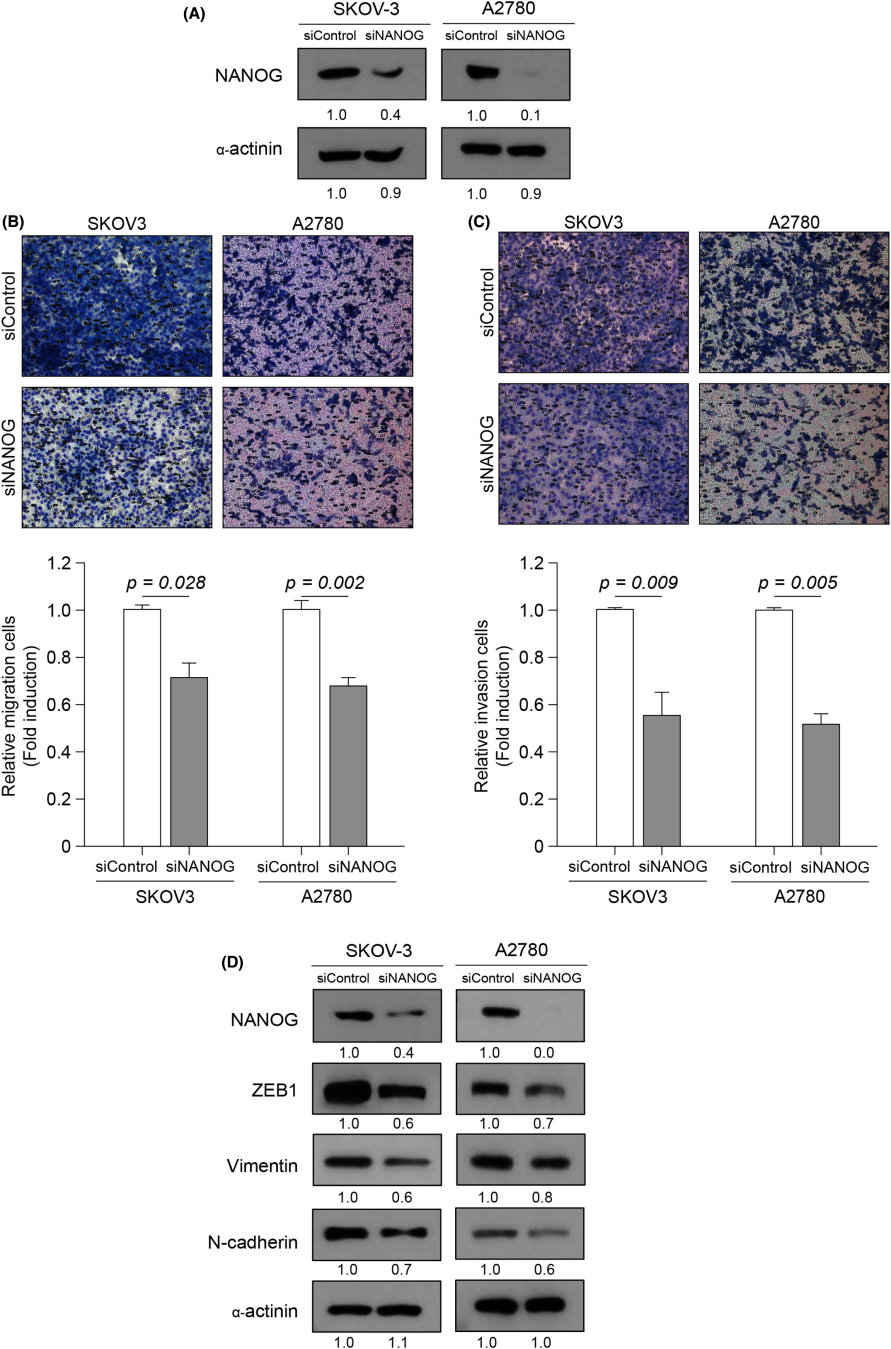
NANOG knockdown inhibits EMT in ovarian cancer cells. NANOG was knocked down in SKOV‐3 and A2780 cells for 48 h. (A) Protein expression of NANOG and α‐actinin was analysed by western blot (numbers below each blot are densitometric values). (B, C) Cell migration and invasion assays were conducted using a Boyden chamber assay. Upper panel: representative image of a Boyden chamber assay. Lower panel: quantitative result of a Boyden chamber assay. (D) Protein expression of NANOG, ZEB1, vimentin, N‐cadherin and α‐actinin was analysed by western blot (numbers below each blot are densitometric values). Error bars represent the mean ± standard error (S.E) of triplicate experiments.

As tumour cells have been shown to acquire drug resistance, metastatic ability, and stem cell traits via the EMT mechanism,^33^ we examined the effects of NANOG expression on the invasion properties and expression of markers associated with EMT in the ovarian cancer malignant cell lines SKOV‐3 and A2780. Cells transfected with two individual siRNAs or pools of siRNAs were monitored for NANOG protein levels by Western blot analysis 48 h post‐transfection. siRNA pools for NANOG significantly reduced NANOG protein expression and were therefore used for subsequent analyses (Figure S1A and Figure 3A). Boyden chamber assays revealed significantly reduced migration (Figure 3B) and invasion (Figure 3C) in siNANOG‐transfected SKOV‐3 and A2780 cells compared with the control. In addition, ZEB1, EMT‐related transcription factors, and mesenchymal markers, such as vimentin and N‐cadherin, were suppressed after NANOG knockdown (Figure 3D). Similar results were obtained in cells transfected with individual siRNAs (Figure S1B–D). These results suggest that NANOG promotes migration and invasion of ovarian cancer cells and the EMT process.

### 3.4 NANOG induces ovarian cancer cell migration and invasion by inhibiting the AMPK/mTOR signalling pathway

**FIGURE 4:**
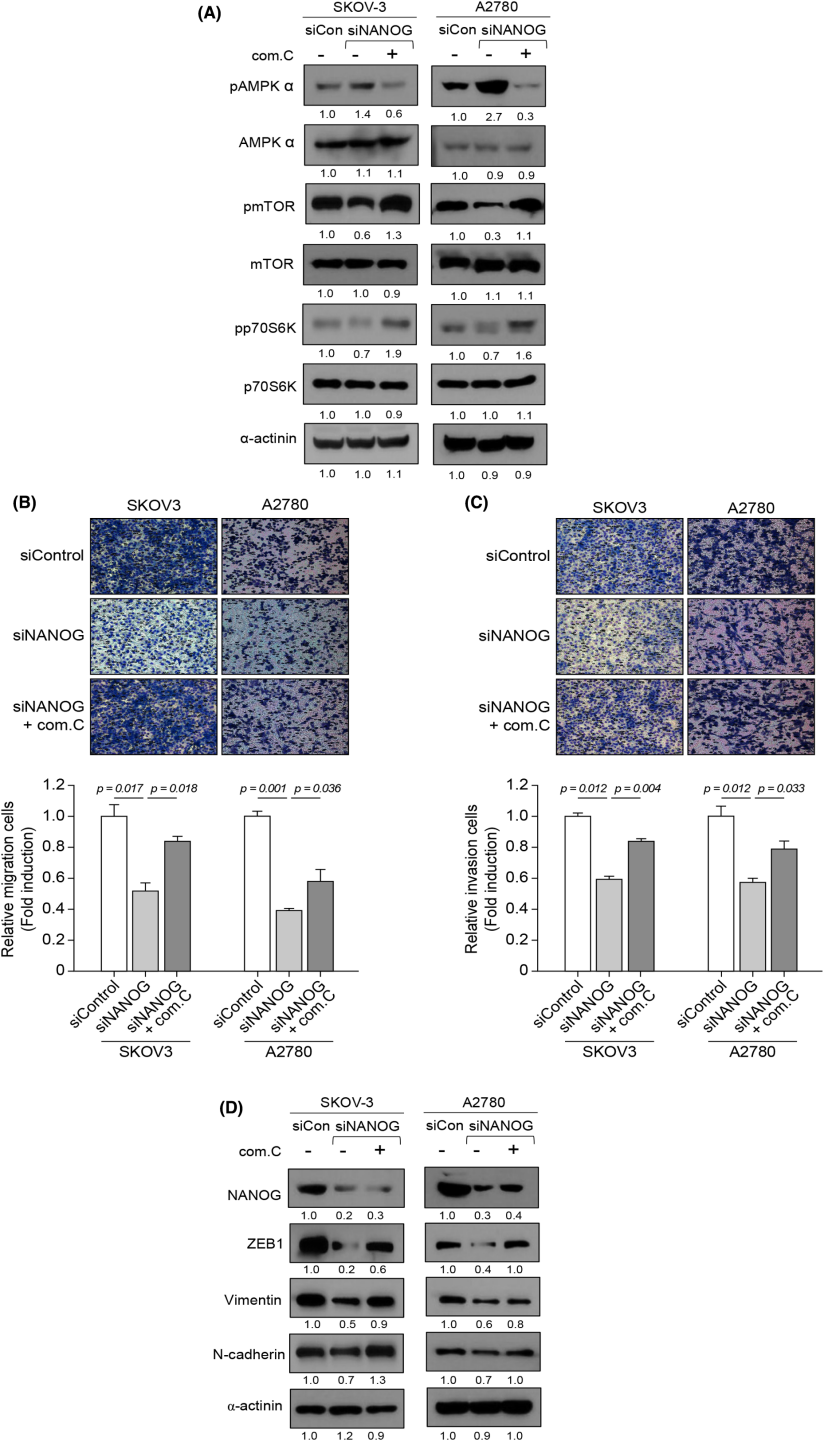
Inhibition of the AMPK signalling pathway reverses the effect of NANOG knockdown on EMT. SKOV‐3 and A2780 cells were transfected with siRNA against NANOG for 48 h followed by treatment with 20 μM compound C for 24 h. (A) Protein expression of pAMPKα, AMPKα, pmTOR, mTOR, pp70S6K, p70S6K and α‐actinin was analysed by western blot (numbers below each blot are densitometric values). (B, C) Cell migration and invasion assays were conducted using a Boyden chamber assay. Upper panel: representative image of a Boyden chamber assay. Lower panel: quantitative result of a Boyden chamber assay. (D) Protein expression of NANOG, ZEB1, vimentin, N‐cadherin and α‐actinin was analysed by Western blot (numbers below each blot are densitometric values). Error bars represent the mean ± standard error (S.E) of triplicate experiments.

Given that NANOG expression stimulates ovarian cancer cell invasion and migration, we subsequently explored the AMPK/mTOR signalling pathway, which plays an important role in tumour development and metastasis. In addition, AMPK has been observed to exhibit tumour suppressive‐like activity in ovarian cancer cells.^34^ To examine the effects of NANOG on AMPK signalling, we firstly analysed AMPK activation by detecting phospho‐AMPKα (Thr^172^). In SKOV‐3 and A2780 cells, suppression of NANOG expression resulted in decreased phosphorylation of mTOR and p70S6K, a key mTORC1 target, as well as increased phosphorylation of AMPK (Figure 4A). These results demonstrate that NANOG knockdown activates the AMPK/mTOR signalling pathway.

To verify that the effects of NANOG on migration and invasion were mediated by AMPK in ovarian cancer cells, we used the AMPK inhibitor, compound C, in siNANOG‐transfected SKOV‐3 and A2780 cells. When the cells were treated with an AMPK inhibitor (com. C), the phosphorylation of AMPK, mTOR and p70S6K was reversed. The decrease in migration and invasion induced by NANOG knockdown was significantly reversed by treatment with compound C (Figure 4B,C). Furthermore, the AMPK inhibitor enhanced the expression of ZEB‐1, vimentin and N‐cadherin in both cell lines (Figure 4D). These results suggest that knockdown of NANOG in ovarian cancer cells activates the AMPK/mTOR signalling pathway, subsequently inhibiting invasion and migration.

## 4 AICAR SUPPRESSES EMT AND EXPRESSION OF STEMNESS‐RELATED GENES IN OVARIAN CANCER CELLS

**FIGURE 5:**
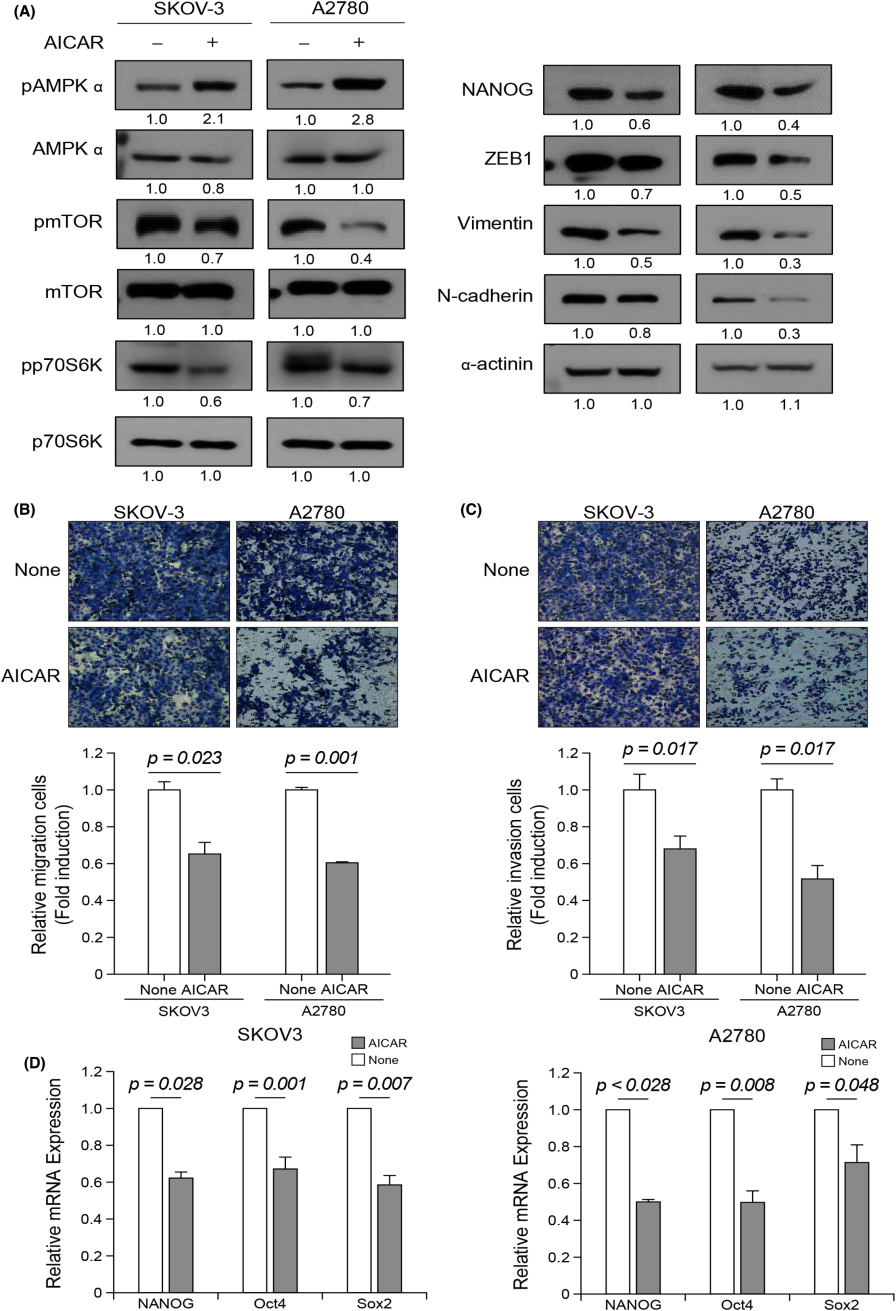
AICAR suppresses EMT and expression of stemness‐related genes. (A) SKOV‐3 and A2780 cells were treated with 1 mM AICAR for 24 h. Protein expression of pAMPKα, AMPKα, pmTOR, mTOR, pp70S6K, p70S6K, NANOG, ZEB1, vimentin, N‐cadherin and α‐actinin was analysed by western blot (numbers below each blot are densitometric values). (B, C) SKOV‐3 and A2780 cells were treated with 1 mM AICAR for 48 h. Cell migration and invasion assays were conducted using a Boyden chamber assay. Upper panel: representative image of a Boyden chamber assay. Lower panel: quantitative result of a Boyden chamber assay. (D) The effect of AICAR on NANOG, Oct4 and Sox2 mRNA expression level was determined by qRT‐PCR. Error bars represent mean ± standard error (S.E) of triplicate experiments.

To further investigate whether AMPK activation is sufficient to suppress cell migration and invasion, and EMT progression in ovarian cancer cells, explored the effect of the AMPK activator AICAR. Treatment with AICAR greatly increased the phosphorylation of AMPK, which was accompanied by decreased phosphorylation of mTOR and p70S6K (Figure 5A). In addition, AICAR suppressed EMT by inhibiting the expression of mesenchymal marker, ZEB‐1, vimentin and N‐cadherin. Boyden chamber assays showed that AICAR‐treated SKOV‐3 and A2780 cells exhibited reduced migration (Figure 5B) and invasion (Figure 5C) compared with untreated cells. Collectively, these data indicate that AICAR may inhibit ovarian cancer cell invasion and migration by targeting EMT.

As AMPK has been shown to be involved in modulating NANOG stability and expression,^32^, ^35^ we also investigated whether AMPK regulates stemness‐related gene expression in ovarian cancer cells. As shown in Figure 5D, AICAR exhibited a significant decrease in mRNA levels of NANOG, Oct4 and Sox2, which is in good agreement with a previous report demonstrating the effects of AICAR on the self‐renewal and differentiation of mES cells.^36^ These data indicate that AMPK activation by AICAR may lead to decreased cancer stemness by regulating the expression of stemness‐related genes such as NANOG, Oct4 and Sox2 in ovarian cancer cells.

## 5 DISCUSSION

The transcription factor NANOG plays a role in embryonic stem cell self‐renewal and is essential for maintaining cancer stem cell properties. The deregulated and abnormal expression of NANOG appears to play a critical role in oncogenesis.^9^ In the present study, we investigated the clinical relevance of NANOG expression in ovarian cancer and the molecular mechanism by which NANOG mediates EMT process. Furthermore, we evaluated the relationship between NANOG and AMPK/mTOR signalling pathway and examined the clinical and prognostic significance of pAMPK in ovarian cancer. To our knowledge, this is the first study to investigate pAMPK expression in a large cohort of EOC patients.

AMP‐activated protein kinase (AMPK), a heterotrimeric complex composed of a catalytic subunit (α) and two regulatory subunits (β and γ), is a metabolic sensor that respond to external stressors by maintaining energy homeostasis. AMPK is phosphorylated and activated by serine/threonine kinase 11 (STK11), also known as liver kinase B1 (LKB1^37^). LKB1 is a well‐known tumour suppressor that was first identified in the Peutz–Jeghers familial cancer syndrome. The LKB1/AMPK pathway has recently been shown to be critically involved in tumour cell migration and invasion by activating numerous signalling pathways and regulating gene expression.^38^ In addition, studies examining the potential relationship between the AMPK subunit and its clinicopathological significance in ovarian cancer revealed that patients with high AMPK α2 expressions had a lower disease recurrence rate and better overall and disease‐free survival rates.^39^ In addition, low expression of AMPK β1 is consistent with the lower activity of AMPK in ovarian cancers that are metastatic, high‐grade and advanced in stage.^34^ In this study, we showed that low expression levels of pAMPK were significantly correlated with poor DFS and OS in EOC. Consistent with our results, many other studies have explored the prognostic value of AMPK in human cancers, including gastric cancer, colorectal cancer, lung cancer, renal cell carcinoma and hepatocarcinoma.^24^, ^25^, ^40^, ^41^, ^42^

EMT is critically involved in tumour invasion, metastasis and resistance to therapy. Therefore, the molecular players involved in this process represent attractive targets in oncology. As NANOG^high^/pAMPK^low^ was revealed to be a more valuable predictive biomarker for DFS and chemotherapy response in EOC patients than single protein expression, we next focused our attention on the mechanism of EMT progression by NANOG and the role of AMPK in this process. Our data showed that siRNA‐mediated NANOG knockdown drastically inhibited cell migration and invasion as well as the EMT process by activating the AMPK/mTOR signalling pathway in SKOV‐3 and A2780 ovarian cancer cells. However, we were unable to elucidate the precise molecular mechanism of AMPK activation by NANOG knockdown in this study. One of the potential hypotheses to explain this mechanism is the effect of the expression of Twist1 and Bmi1, which are well‐known targets of NANOG,^43^, ^44^ on AMPK activity. A recent study demonstrated that silencing of Twist1 triggered ATP depletion, leading to AMPK activation in non‐small cell lung cancer (NSCLC) cells.^45^ In addition, downregulation of Bmi1 was associated with activation of the PKCζ‐AMPK pathway in chronic myeloid leukaemia (CML) cells.^46^ In addition to NANOG, Twist1 and Bmi1 are important factors in the promotion of EMT and are positively correlated with poor prognosis in various cancers.^47^ Thus, further studies are needed to clarify the molecular basis of the regulation of the AMPK signalling pathway by NANOG and its downstream target genes during metastasis.

Recent studies have demonstrated that NANOG is negatively regulated by AMPK. A study reported that AMPK promotes Speckle‐type POZ protein (SPOP)‐mediated NANOG ubiquitination and degradation in prostate cancer.^32^ In mouse embryonic stem cells, treatment with AICAR, an AMPK activator, suppressed both transcriptional and post‐translational expression of NANOG.^36^ Likewise, through pharmacological activation with A769662 or through transfection, AMPK upregulation resulted in reduced expression of stem cell markers, including NANOG, in hepatocellular carcinoma cells.^48^ While the involvement of AMPK in CSC development has not been firmly elucidated, some studies have recently shown reciprocal regulation between AMPK and stemness factors such as NANOG, Oct4 and Sox2. For example, Sox2‐overexpressed breast cancer cells exhibit downregulated AMPK signalling and activated mTOR to maintain their cancer stem‐like phenotypes.^49^ In HCC cells, ectopic expression of the cancer stem cell marker CD90 increases sphere formation, soft agar growth, and tumorigenicity via the AMPK and mTOR pathways.^50^ It has also been reported that sorafenib‐resistant HCC cells have increased tumorigenic potential and show higher expression of stem‐related genes (NANOG, Oct4, CD133 and alpha fetoprotein) and lower levels of AMPK phosphorylation in vitro and in vivo.^48^ Considering the abovementioned research and our results, overexpression of pluripotency factors and suppression of the AMPK signalling pathway in cancer cells are thought to be interconnected. Further studies to elucidate the functional crosstalk between NANOG and AMPK pathways in ovarian cancer cells may result in the identification of therapeutic targets, paving the way for more effective and safe manner. In addition, agents that target CSC‐associated cell surface receptors and signalling pathways have generated promising pre‐clinical results and are currently entering clinical trials. Two novel CSC‐specific small‐molecule multi‐kinase inhibitors, amcasertib (BBI503) and napabucasin (BBI608), demonstrated significant anti‐NANOG activity.^51^ These NANOG inhibitors have been used in clinical trials and were reported to be safe in early phase I studies of advanced, relapsed or recurrent (R/R) solid tumours. There is an ongoing phase II clinical trial with NANOG inhibitor in combination with sorafenib in adult patients with hepatocellular carcinoma (Clinical trial ID: NCT02279719).^52^

In mammals, rapamycin (mTOR) targets a serine/threonine kinase that is involved in the proliferation and growth of cells.^53^ Recent studies have indicated that in the majority of patients with EOC, mTOR is frequently turned on and is correlated with poor survival rate.^54^ Furthermore, in approximately 70% of EOC patients, phosphatidylinositol 3‐kinase (PI3K), an upstream positive regulator of mTOR, is activated, resulting in the hyperactivation of PI3K/mTOR signalling.^3^ With the approval of the mTOR complex 1 (mTORC1) inhibitors Everolimus and Temsirolimus for breast cancer and renal cell carcinoma,^55^, ^56^ several inhibitors of PI3K/mTOR have been widely developed and clinical trials have been evaluated. Several studies have been conducted in Phase I/II clinical trials, showing that mTOR inhibitors exhibit more promising results in combination with anti‐angiogenics and/or chemotherapeutic agents than as a monotherapy.^57^ Phase I/II clinical trials are currently underway to investigate dual‐PI3K/mTOR inhibitors in combination with chemotherapy or targeted therapies in breast cancer, renal cell carcinoma and prostate cancers and have promising results that may be useful for ovarian cancer trials in the future.^58^ Since the PI3K/mTOR signalling pathway is recognized as being crucial for therapeutic interventions in many cancers, including EOC, an understanding of the molecular mechanisms that regulate PI3K/mTOR signalling is required. Several studies have established that AMPK is an upstream regulator that modulates mTOR.^17^ Consistent with these studies, our results showed that pharmacological inhibition of AMPK by compound C in siNANOG‐transfected ovarian cancer cells increased the phosphorylation of mTOR and p70S6K and reversed the effects of NANOG knockdown. Furthermore, AICAR, an AMPK activator, suppressed the migration and invasion of ovarian cancer cells through an AMPK/mTOR‐dependent pathway. These findings implicate AMPK as a potential therapeutic target in EOC.

In conclusion, our findings established that the combination of high NANOG and low pAMPK expression is associated with EOC progression and platinum resistance, suggesting a potential predictive biomarker for clinical management in EOC patients. Functionally, knockdown of NANOG in ovarian cancer cell lines hindered cell migration and invasion, as well as the EMT process via the AMPK/mTOR signalling pathway. Further exploration is required to understand the underlying mechanistic link between NANOG and AMPK in EOC. Finally, the present study demonstrated that the AMPK signalling pathway regulates pluripotency factor expression (Sox2, Oct4 and Nanog) in ovarian cancer cells, suggesting that strategies targeting AMPK might provide a novel approach to control the cancer stem cells of EOC patients.

## AUTHOR CONTRIBUTIONS

**Hee Yun:** Formal analysis (equal); writing – original draft (equal). **Gwan Hee Han:** Investigation (equal). **Julie Kim:** Investigation (equal). **Joon‐Yong Chung:** Investigation (equal). **Jae‐Hoon Kim:** Investigation (equal). **Hanbyoul Cho:** Conceptualization (equal); writing – review and editing (equal).

## FUNDING INFORMATION

This work was supported by the National Research Foundation of Korea (NRF) grant funded by the Korea government (MIST) (NRF‐2020R1A2C2004782). This research was supported by the Bio & Medical Technology Development Program of the National Research Foundation (NRF) funded by the Korean government (MSIT) (NRF‐2017M3A9B8 069610). This study was also supported by a faculty research grant of Yonsei University College of Medicine (No. 6–2020‐0226).

## CONFLICT OF INTEREST

The authors confirm that there are no conflicts of interest.

## INSTITUTIONAL REVIEW BOARD STATEMENT

All biological samples were collected after obtaining informed consent from participants, following the guidelines of the institutional review board (IRB) of Gangnam Severance Hospital (IRB No. 3‐2020‐0377).

## Supporting information


Figure S1

## Data Availability

Data available on request from the authors.
