# Supplementary material for: NANOG regulates epithelial–mesenchymal transition via AMPK/mTOR signalling pathway in ovarian cancer SKOV‐3 and A2780 cells
Source: J Cell Mol Med. 2022 Sep 16;26(20):5277–91. doi: 10.1111/jcmm.17557 (PMC9575063; doi:10.1111/jcmm.17557)
Supplement: Supplementary file 1 — Figure S1 [file JCMM-26-5277-s001.docx]

Supplementary Figure


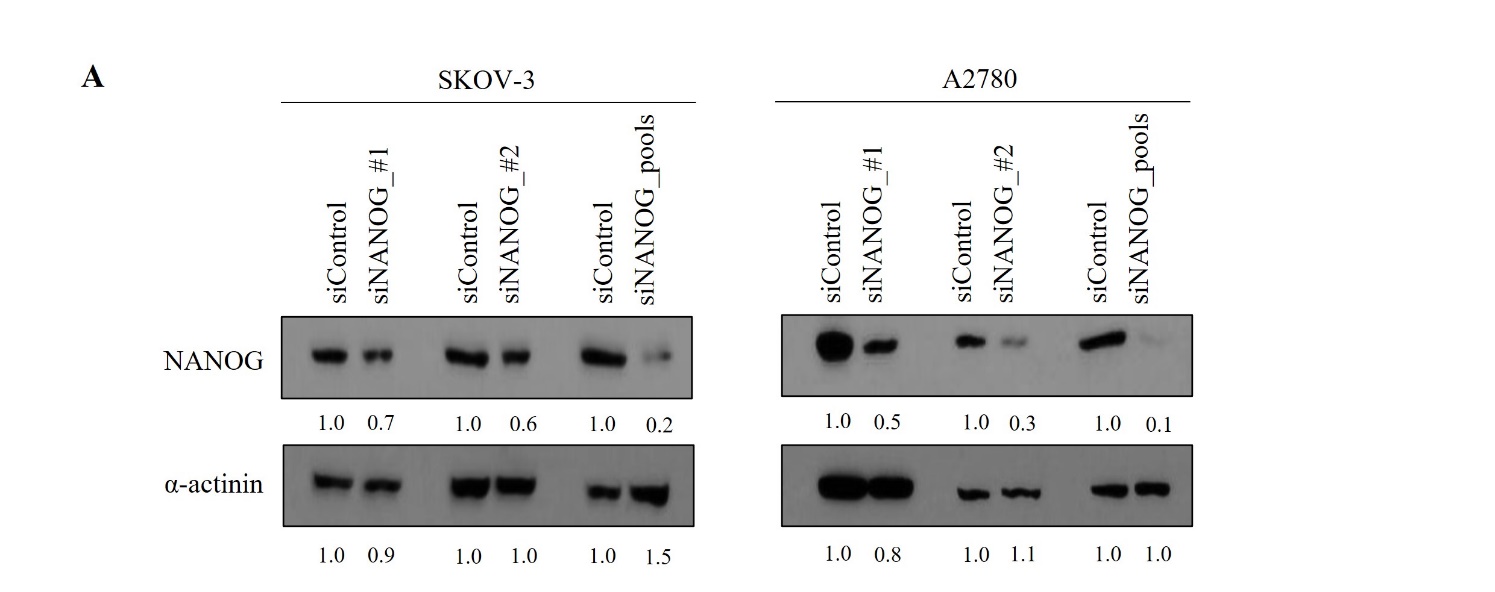


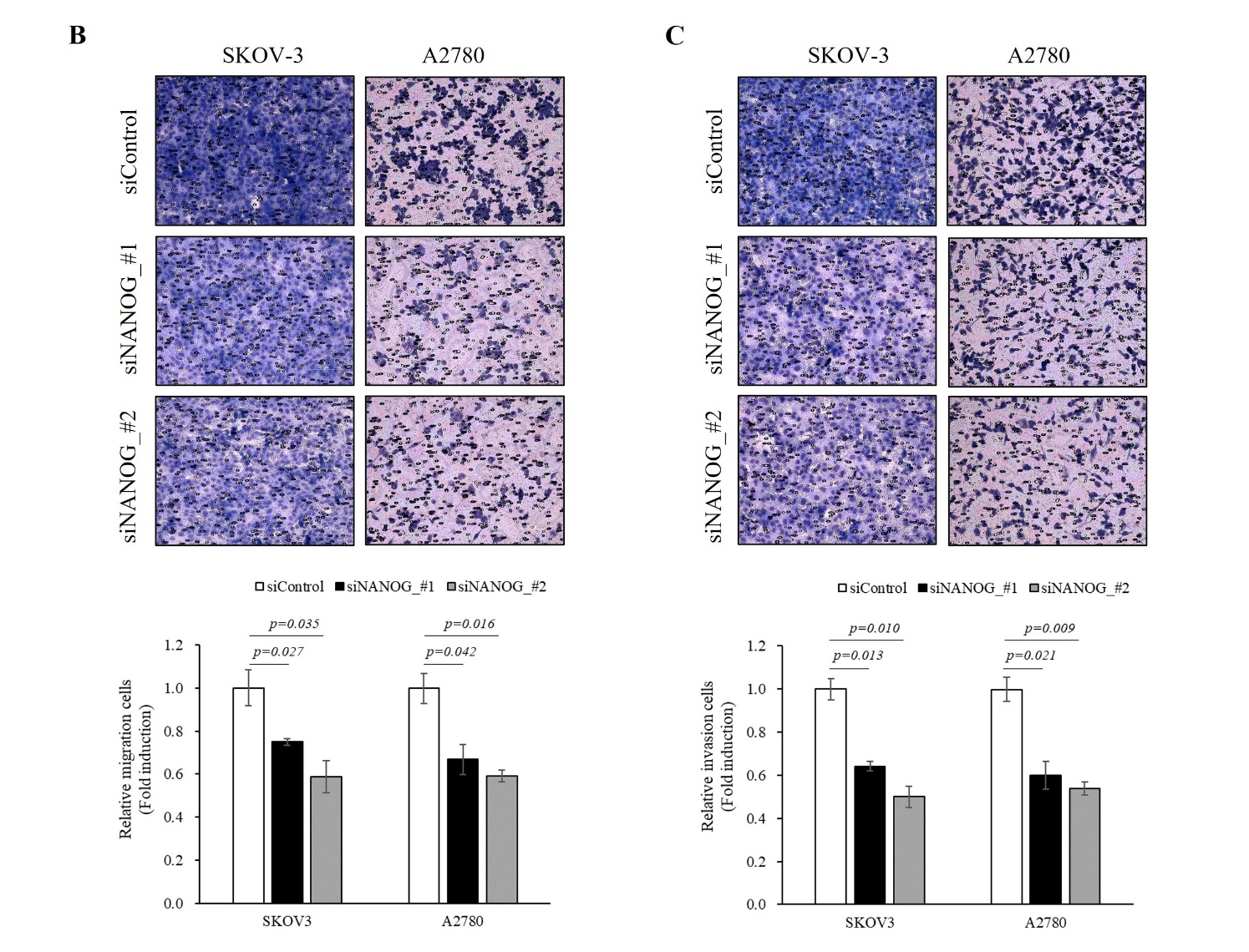


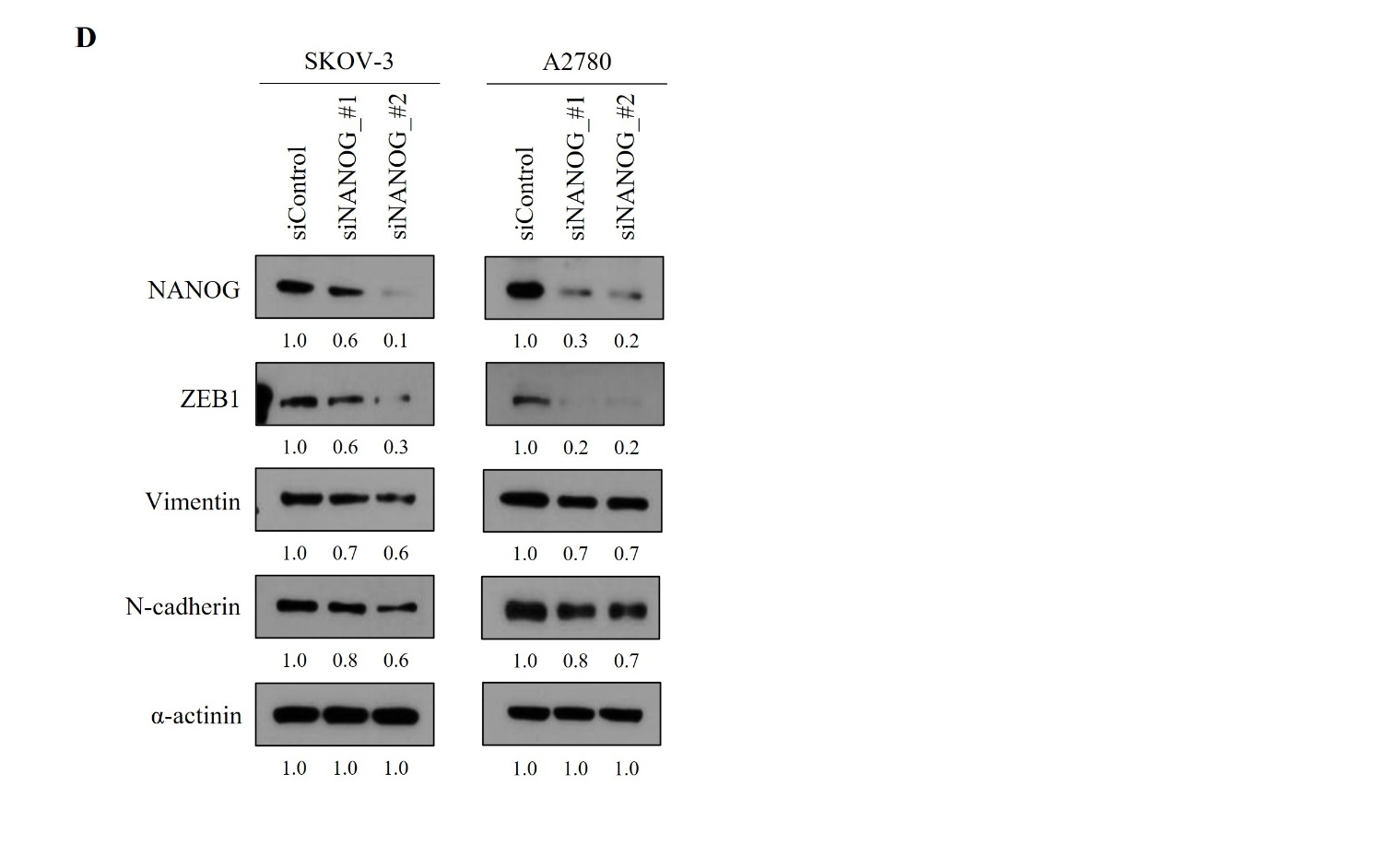


**Supplementary Figure 1.** (A) SKOV-3 and A2780 cells were transfected with two individual siRNAs or pools of siRNAs against NANOG for 48 h. Protein expression of NANOG and $\alpha$-actinin was analyzed by western blot (numbers below each blot are densitometric values). (B, C) Cell migration and invasion assays were conducted using a Boyden chamber assay. Upper panel: representative image of a Boyden chamber assay. Lower panel: quantitative result of a Boyden chamber assay. (D) Protein expression of NANOG, ZEB1, vimentin, N-cadherin and $\alpha$-actinin was analyzed by western blot (numbers below each blot are densitometric values). Error bars represent the mean ± standard error (S.E) of triplicate experiments.
